# H_2_S gasotransmitter-responsive polymer vesicles†
†Electronic supplementary information (ESI) available. See DOI: 10.1039/c5sc03576g


**DOI:** 10.1039/c5sc03576g

**Published:** 2015-12-03

**Authors:** Qiang Yan, Wei Sang

**Affiliations:** a Department of Macromolecular Science , Key Laboratory of Molecular Engineering of Polymers of the Education Ministry of China , Fudan University , Shanghai , China 200433 . Email: yanq@fudan.edu.cn

## Abstract

Building biomimetic polymer vesicles that can sense a biological signaling molecule is a tremendous challenge at the cross-frontier of chemistry and biology. We develop a new class of *o*-azidomethylbenzoate (AzMB)-containing block copolymer that can respond to an endogenous signaling molecule, hydrogen sulfide (H_2_S). Such a gasotransmitter can trigger cascade chemical reactions to sever the AzMB side functionalities, which alters the polymer amphiphilicity and further leads to a controllable disassembly of their self-assembly vesicular nanostructure. Moreover, if we introduce cystathionine γ-lyase (CSE), a specific enzyme converting cysteine into H_2_S, onto the vesicle membrane, the polymersomes can extend their responsive scope from H_2_S to a specific amino acid bioactivator. We anticipate that this polymer model could open up a new avenue for constructing biosignal-triggered nanocapsules for intracellular applications.

## Abstract 2

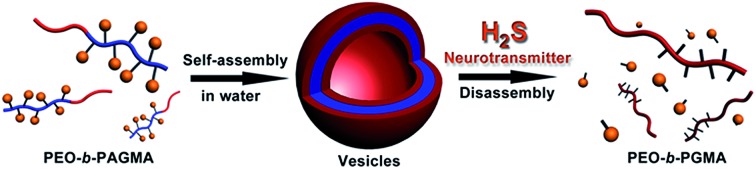
A type of new polymeric vesicle self-assembly by *o*-azidomethylbenzoate-containing diblock copolymer can respond to the cell signaling molecule hydrogen sulfide (H_2_S). The intracellular H_2_S gasotransmitter can trigger biomimetic polymersome disruption for targeted drug delivery.

## Introduction

Stimuli-responsive polymer vesicles, regarded as a kind of smart nanovehicle, have sparked great attention in recent years since they are a promising prospective candidate for drug delivery systems and nanomedicine.^1^ In general, these nanovehicles can respond to external stimuli and then undergo drastic chemical structural changes,^2^ thus inducing controlled dissociation so as to release cargos for target therapy. To date, a variety of external signals including temperature,^3^ light,^4^ and electric field,^5^ as well as physiological factors, such as redox,^6^ pH,^7^ and enzyme^8^ have been exploited for building responsive polymersomes. Recently, more and more intracellular applications have required us to directly utilize bioactive molecules as triggers to manipulate payload release. This new strategy has two particular advantages: endogenous stimuli are conducive to enhancing specific drug biodistribution in diseased cells1*a* and prevent cell damage from chemical agent accumulation. In this respect, despite an imperative to simulate bioresponsive modes, building macromolecular assemblies that can sense a particular bioactivator remains elusive.^9^

Hydrogen sulfide (H_2_S), known as a toxic gas in the atmosphere, is also an important neuromodulator and cell signaling molecule. In the cell, H_2_S can be generated from l-cysteine *via* cystathionine γ-lyase (CSE) mediated decomposition.^10^ The latest reports have demonstrated that it plays a pivotal role in tuning blood vessel dilation, resisting inflammation and inducing cell apoptosis.^11^ Metabolic disturbance of H_2_S is directly related to numerous diseases including angiocardiopathy and neurodegeneration. Taking into account the importance of the effect of H_2_S on cells, exploring how to use this bioactivator as a stimulation mode to activate polymer self-assembly behaviors for targeted drug delivery is quite challenging.

**Fig. 1:**
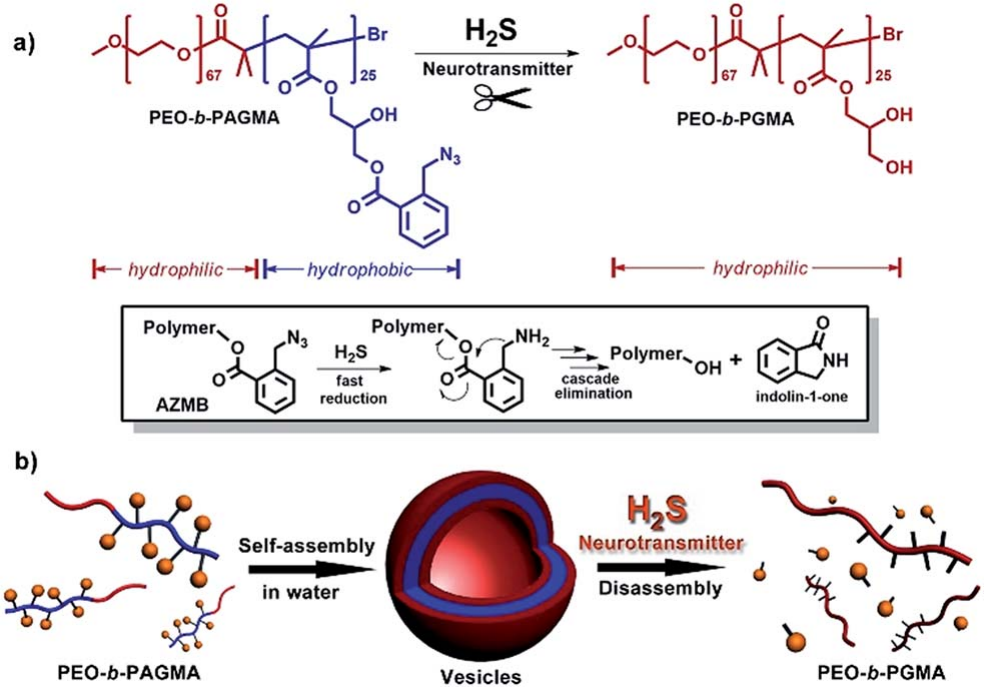
(a) H_2_S-responsive cleavage of *o*-azidomethylbenzoate (AzMB)-containing diblock copolymer (PEO-*b*-PAGMA) and H_2_S-induced cascade reaction mechanism. (b) Schematic illustration of their polymer self-assembly into vesicles and H_2_S-responsive controlled disassembly process.

At present, some nascent studies have been devoted to embedding biosignals into polymer systems. A successful example is the proposed CO_2_-sensitive polymer assemblies of Yuan and Zhao *et al*.^12^ The Davis group designed a nitric oxide (NO)-tuning copolymer.^13^ In spite of the progress at this frontier, polymers that can feedback H_2_S gasotransmitter have not been synthesized so far. Here we developed the new idea of using a class of peculiar *o*-azidomethylbenzoate-containing block copolymers to fabricate H_2_S-responsive polymer vesicles. Their disassembly process is dependent on the stimulus concentration. Actually, *o*-azido-methylbenzoate (AzMB) is a sensitive self-cleavable precursor, which can be converted into benzylamine by H_2_S, and the latter is able to cause a series of intramolecular cascade reactions that finally sever the benzoyl bond (Fig. 1a). Based on this, we speculated that the AzMB pendants covalently linked to the copolymer could be cleaved by a low concentration of H_2_S. This will thereby drive a change in amphiphilicity of the block copolymer, further triggering a desirable disassembly of the polymer aggregates, as shown in Fig. 1b.

## Results and discussion

### Designing a H_2_S-responsive block copolymer

To fulfil the goal, we designed and synthesized a kind of diblock copolymer, consisting of a biocompatible poly(ethylene oxide) (PEO) chain as the hydrophilic block and a poly(*o*-azidomethyl benzoyl glycerol methacrylate) chain (termed as PAGMA) as the H_2_S-sensitive hydrophobic segment. This target copolymer, PEO-*b*-PAGMA, was prepared by atom transfer radical polymerization with a well-defined molecular weight (*M*_w_ = 11.2 kDa) and near-monodispersion (*M*_w_/*M*_n_ = 1.09, the details of the synthesis and characterization are in the ESI†).

**Fig. 2:**
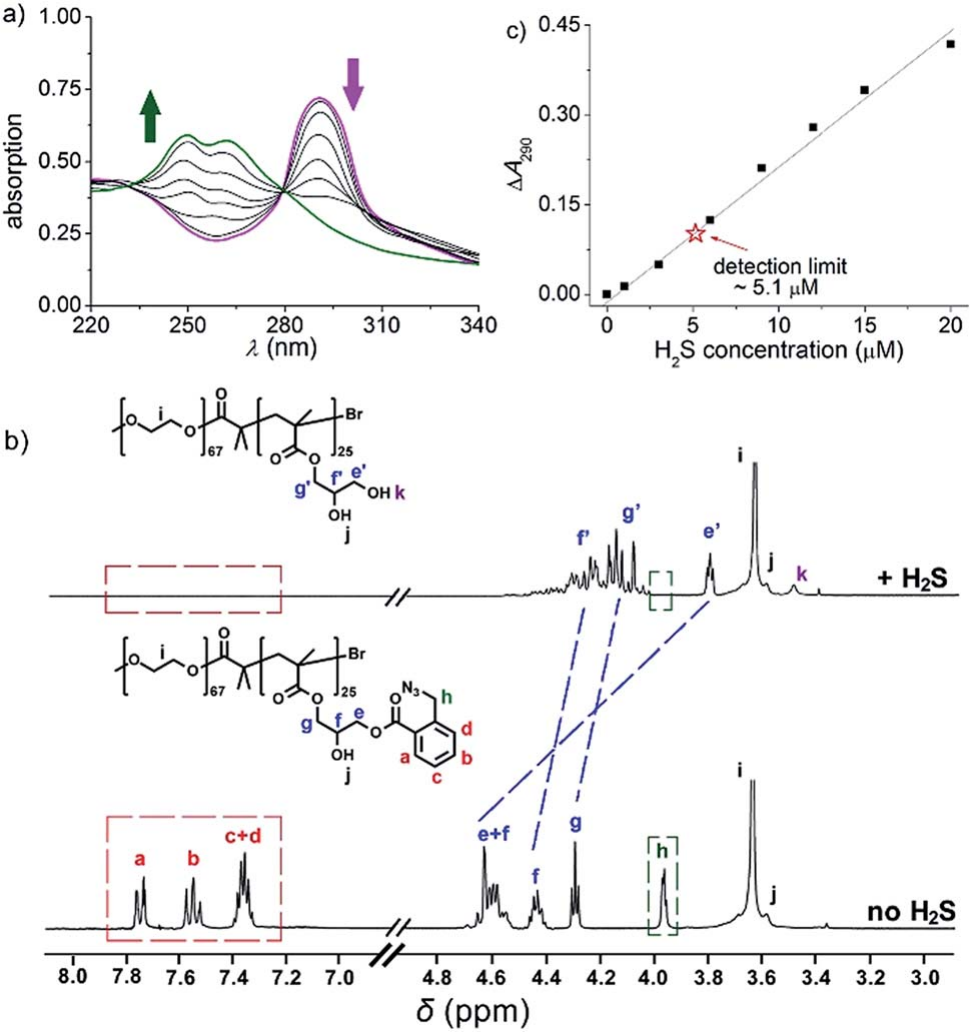
(a) UV-vis absorption changes of PEO-*b*-PAGMA under different H_2_S concentrations (from pink to green curve): 0, 2, 4, 6, 10, 12, 16, and 20 μM. (b) ^1^H NMR spectra comparison of PEO-*b*-PAGMA before and after H_2_S treatment (*d*_6_-DMSO). (c) The UV-vis absorbed intensity changes of PEO-*b*-PAGMA for different H_2_S levels (*λ*_max_ = 290 nm). All the experiments were carried out at the polymer concentration of 9 × 10^–3^ g L^–1^, containing 20 μM AzMB groups.

We investigated the H_2_S-responsiveness of this copolymer using UV-vis spectroscopy. While increasing H_2_S concentration, the absorption changes of PEO-*b*-PAGMA can be monitored. We found that in the absence of an external stimulus, the polymer solution (9 × 10^–3^ g L^–1^, containing 20 μM concentration of AzMB groups) exhibited a characteristic absorption at 290 nm ascribed to the AzMB species (Fig. 2a, pink curve). Upon gradual addition of H_2_S from 0 to 20 μM, interestingly, the intensity of the AzMB absorption band declined by 94%, whereas a group of new double peaks at 248 nm and 259 nm appeared and slowly strengthened (Fig. 2a, green curve), suggesting that H_2_S can react with the AzMB functionality. To further elucidate this chemical process, before and after gas treatment, all the reactive components in solution were separated by liquid chromatography and analysed by ^1^H NMR spectroscopy (Fig. 2b). Before the stimulus, it is clear that the ^1^H NMR spectrum of PEO-*b*-PAGMA showed a group of typical aromatic region proton shifts (H_a_–H_d_, *δ* = 7.30–7.76 ppm) and a benzyl peak (H_h_, *δ* = 3.95 ppm) ascribed to AzMB species. After the copolymer solution was exposed to H_2_S, however, all the above peaks in the final products totally vanished, indicating the cleavage of the benzoyl bond. In addition, the three groups of methylene peaks (H_e_, H_f_ and H_g_) belonging to the glycerol motif are relatively shifted upfield (H_e_ → H_e′_: *δ* = 4.63 → 3.81, H_f_ → H_f′_: *δ* = 4.43 → 4.36, H_g_ → H_g′_: *δ* = 4.31 → 4.19), which is in line with the spectrum of poly(glycerol methacrylate) (PGMA, Fig. S3 in ESI†). These results provided key evidence of the detachment of AzMB pendants from the copolymer main chain. Furthermore, we attempted to reveal the reaction mechanism of H_2_S cutting off the AzMB group. To this end, we separated and purified the other byproducts from the reactive solution. A cyclic benzolactam compound was detected in the filtrate residue. Its ^1^H NMR spectrum perfectly matched with commercial indolin-1-one (Fig. S4 in the ESI†) and its UV-vis absorbance was in accord with the double-peak curve (Fig. S5 in ESI†), indicating that H_2_S can facilitate an intramolecular cyclization of the benzoyl bond to yield an indolin-1-one byproduct.

Infrared spectra (IR, Fig. S6 in ESI†) corroborated this reactive mechanism: the initial PEO-*b*-PAGMA solution displayed a strong azido group stretching vibration (2102 cm^–1^); at 6 min of the H_2_S gas stimulus, the benzylazide peak was greatly depressed but a shoulder peak at 3465 cm^–1^ ascribed to benzylamine was reinforced. However, this new vibration band vanished after 30 min of H_2_S treatment and the final PEO-*b*-PGMA was generated, as indicated by the disappearance of the phenyl group (1614 cm^–1^) and the appearance of the broad hydroxyl band (3240–3640 cm^–1^). All these findings demonstrate that H_2_S quickly transforms benzylazide into a highly reactive nucleophilic benzylamine intermediate, and the latter is capable of attacking intramolecularly on the adjacent benzoyl to induce a cascade self-elimination reaction, leading to a site-specific chemical scission (Fig. 1a).

Since the designed PEO-*b*-PAGMA can respond to H_2_S, we next aimed to quantitatively test its sensitivity. If the detection limit of H_2_S is defined as a 10% change in UV-vis absorbance,^13^ it is worth noting that PEO-*b*-PAGMA is determined to be extremely sensitive to the H_2_S stimulus (detection limit is 5.1 μM, Fig. 2c). Recent studies have indicated that the H_2_S concentration in the brain is likely less than 9.2 μM,^14^ but is nonetheless capable of activating the cleavage reaction. Additionally, we discovered that this polymer system showed a linear H_2_S concentration-dependent responsiveness, which means that the cleavage ratio of the polymer is adjustable.

### H_2_S-triggered controlled disassembly of the polymersomes

**Fig. 3:**
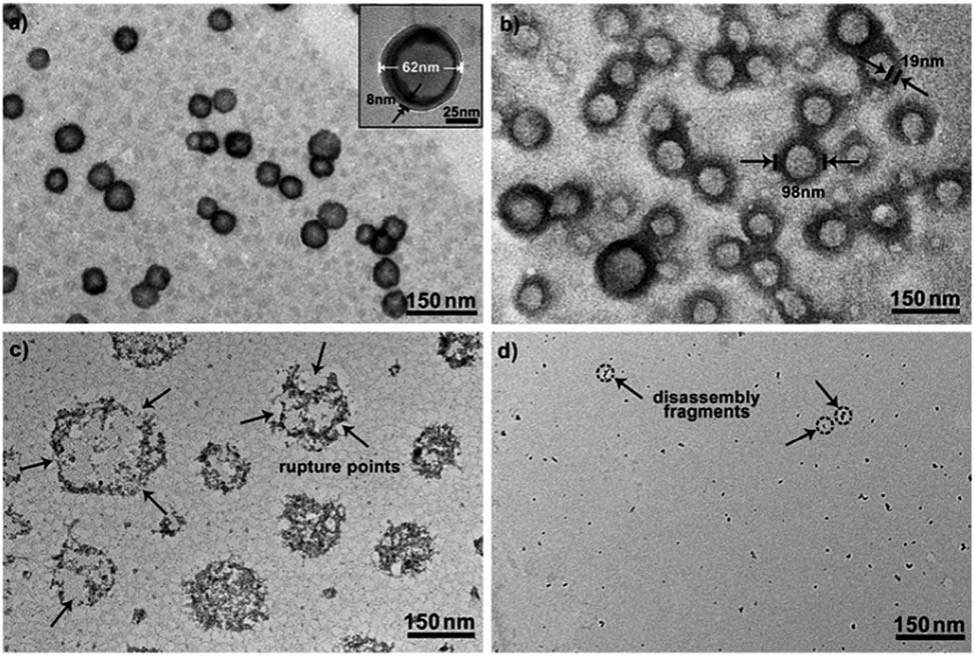
TEM images of PEO-*b*-PAGMA aggregates for various levels of H_2_S stimulus: (a) 0 μM, (b) 15 μM, (c) 30 μM and (d) 45 μM (the polymer concentration is 0.02 g L^–1^, which contains an AzMB group concentration of 45 μM).

To further explore whether this polymer system is adaptable for use as a drug nanovehicle, we studied its self-assembly behavior. Owing to the amphiphilicity, PEO-*b*-PAGMA can spontaneously form aggregates in aqueous solution. The critical aggregate concentration (CAC) is determined to be 0.02 g L^–1^, as measured by a fluorescent probe method (Fig. S7 in ESI†).^15^ A transmission electron microscope (TEM) was employed to visualize the size and morphology of these polymeric aggregates. It is clear that PEO-*b*-PAGMA can self-assemble into a sphere-like nanostructure (Fig. 3a). The legible contrast between the dark periphery and hollow center indicates that these globular aggregates have a typical vesicular architecture and their membrane thickness is evaluated to be 8 nm by TEM statistics (Fig. 3a, inset). Normally, in the vesicles, PEO block chains serve as the hydrophilic outer and inner layers while the PAGMA portion is the hydrophobic core layer. The TEM images showed that the average diameter of these aggregates is 62 nm, corresponding to a hydrodynamic radius (*R*_h_) of 34.6 nm determined by dynamic light scattering (DLS, Fig. S8 in ESI†). The small deviation (7.2 nm) between the TEM and DLS results is representative of the fact that DLS measures the hydrated state while TEM, using dried samples, does not. In the absence of any stimuli, these aggregates are stable in water, and neither their size nor shape changes over two months.

**Fig. 4:**
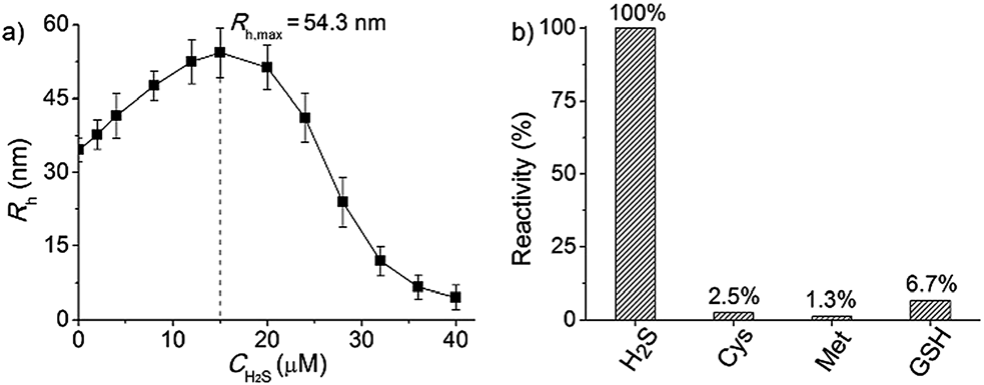
(a) The polymersome radius changing as a function of H_2_S concentration monitored by DLS counting (after gas treatment for 30 min, the polymer solution was incubated for 30 min and then tested by DLS). (b) The responsive specificity comparison among other sulphur-containing bioactivators (H_2_S: 20 μM; Cys, Met and GSH: 45 μM; H_2_S is the reference of 100% activity and the treatment time of all stimulants was kept at 60 min).

Since the PAGMA block chain can react with H_2_S and further remove the AzMB moieties to yield PGMA block, we speculated that their polymer assemblies could disassemble in a H_2_S atmosphere. As expected, when H_2_S was passed through the polymer solution (0.02 g L^–1^, containing 45 μM of AzMB groups), their assembling structures began to change. In the presence of a small amount of H_2_S gas (15 μM), these vesicles expanded remarkably. As shown in Fig. 3b, much larger vesicles were dominant in the solution. Their diameter rose on average 160% from 62 nm to 98 nm and their volume increased 410%. The reason is that one-third of the hydrophobic PAGMA block chains convert to hydrophilic PGMA block, which results in a strong hydration effect within the core layer; as a consequence, these vesicles swell quickly in order to lower the interaction free energy. A near double thickening of these vesicle walls from 8 nm to 19 nm supported this hydration-driven expansion mechanism. Similar results have been found in other responsive vesicles.12a,c When the H_2_S level was increased to 30 μM, a large amount of PAGMA block chains transformed. Because the sharp enhancement of the water solubility of the polymer chains resulted in an insupportable interfacial tension, these swollen vesicles started to crack and their membrane presented many random fractured points (Fig. 3c). With injecting H_2_S to a concentration of 45 μM, the majority of AzMB groups were disconnected and the amphiphilic PEO-*b*-PAGMA changed to water-soluble PEO-*b*-PGMA, finally leading to complete vesicle dissociation. Only a few nanofragments remained in solution, whose size (<10 nm) was in line with that of dried PEO-*b*-PGMA unimers (Fig. 3d). DLS monitoring confirmed this disassembly process. By changing the H_2_S aeration time, we could tune the H_2_S stimulus level. Under a lower H_2_S level (15 μM), the *R*_h_ of these aggregates climbed up to 54.3 nm, consistent with the early vesicle expansion. Then their *R*_h_ underwent an abrupt decrease from a maximum value to 4.3 nm with a H_2_S increase to 45 μM, corresponding to the later vesicular burst process (Fig. 4a).

On the other hand, one may doubt that other factors such as mechanical effects could bring about this vesicle disruption. In a control experiment, we prepared a copolymer counterpart, PEO-*b*-poly(benzoyl glycerol methacrylate) (PEO-*b*-PBGMA) lacking the azido group. It can form an analogous nano-object in aqueous solution as well but is unable to disaggregate even at a higher H_2_S level (100 μM), thus eliminating this possibility and proving that the vesicular disassembly mechanism arises from H_2_S-sensitive macromolecular structural alteration (Fig. S9 in ESI†).

### The specificity of H_2_S-triggered polymersome disassembly

To better adapt to the cell environment, it is desirable that this disassembly is H_2_S specific and selective. In biological cells, besides the H_2_S gasotransmitter, there are other sulphur-containing bioactivators to guide cell activities such as cysteine (Cys), methionine (Met), and glutathione (GSH).^16^ According to the above experiments, we know that the cleavage of the polymer leads to an absorption change. Based on this characteristic, we surveyed the effects of these sulphur-containing chemicals by UV-vis spectroscopy. A higher level of bioactivators (45 μM) was injected into the polymer solution and the system was incubated for 60 min. Regarding the H_2_S-induced spectral change as a 100% active reference, other bioactivators (Cys, Met and GSH) showed negligible activities (<7%, Fig. 4b and S10 in ESI†). This shows that these polymersomes entering the cells can selectively respond to the H_2_S gasotransmitter but prevent the influence of other sulphur interferents.

### Extending the bioresponsive scope of the polymersomes

**Fig. 5:**
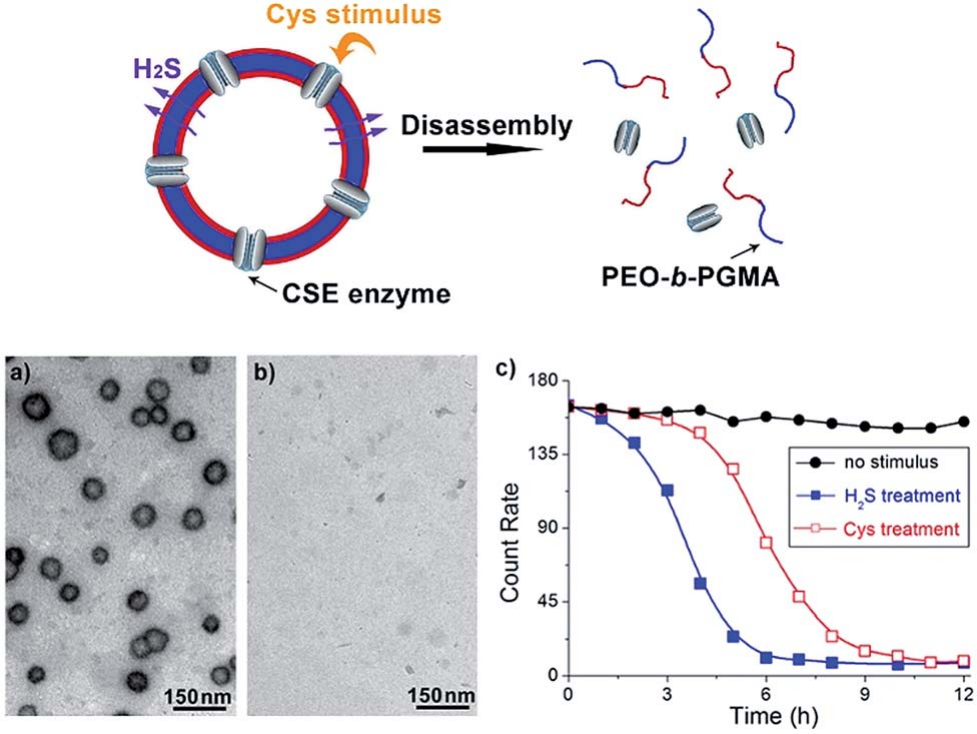
Schematic illustration of Cys-responsive polymer vesicle disruption (top). (a) and (b) TEM images showing Cys-triggered disassembly of CSE/PEO-*b*-PAGMA hybrid polymersomes: (a) no stimulus, (b) injecting 45 μM Cys into the aggregate solution (the polymer concentration is 0.02 g L^–1^, containing an AzMB group concentration of 45 μM). (c) The disassembly rate of the polymersomes upon various conditions: no stimulus (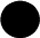), H_2_S treatment (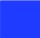) and Cys treatment (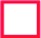). The test was conducted using the DLS counting rate.

In view of the excellent H_2_S-responsiveness of our vesicles, we expected that we could extend their responsive range to a variety of biomolecules or biological metabolites. In the cell, H_2_S can be endogenously produced by many kinds of proteases. For example, CSE enzyme is capable of converting intracellular Cys into H_2_S.^10^ Therefore, introducing CSE into the vesicle system can broaden the responsiveness from H_2_S to a specific amino acid, Cys. Based on this principle, a trace amount of CSE protein (20 nM) was co-assembled with PEO-*b*-PAGMA. After the vesicle forms, there should be a part of CSE dispersed in the solution. To remove these residues, the aggregate solution was centrifuged for 5 min at 11 000 rpm at certain intervals (2 h), and 2 mL of the supernatant was withdrawn and replaced by fresh medium. Measuring the UV-vis spectra of the supernatant until there was no obvious CSE absorption indicated that unassembled CSE was removed and only 35% of proteins were noncovalently anchored on the vesicular membrane (Fig. S11 in ESI†). Adding Cys (45 μM) in place of H_2_S, this amino acid can penetrate across the membrane and further be decomposed into H_2_S by CSE. Afterwards the generated H_2_S can *in situ* react with PAGMA block chains to provoke a similar vesicular disruption (Fig. 5a and b). In comparison to the H_2_S stimulus, the Cys-triggered disassembly rate is 45% slower than that of H_2_S treatment, as indicated by DLS counting experiments (Fig. 5c). It is understandable since the CSE-mediated enzymatic reaction needs an induction period for Cys transmembrane penetration and H_2_S production. In a similar way, if one can introduce other H_2_S-producing proteins into the polymer vesicle, the responsive scope will be expanded to the corresponding biomolecules.

### H_2_S-responsive controlled drug release

Finally, to assess the feasibility of these polymersomes as drug delivery vectors, we performed payload release tests. It is known that the overproduction of H_2_S gasotransmitter can result in blood vessel overexpansion and hypotension.11*a* Epinephrine (EP), as a water-soluble vasoconstrictor, is used for resisting vessel dilation. By encapsulating EP into our vesicles, it is anticipated that when these drug-loaded nanocarriers enter H_2_S-overproducing cells, they can rapidly rupture to liberate internal cargos for *in situ* inhibition of vessel dilation.

**Fig. 6:**
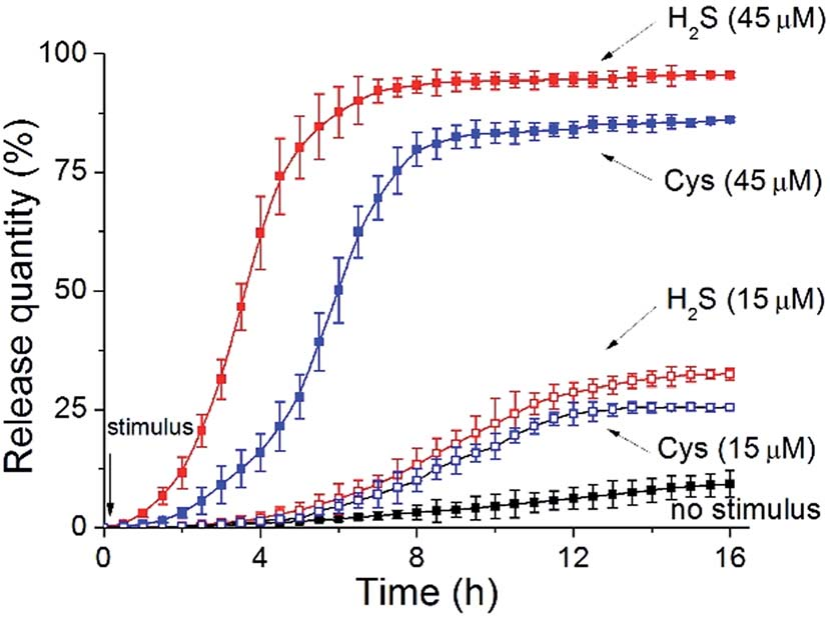
Controlled drug release of EP from PEO-*b*-PAGMA vesicles or CSE/PEO-*b*-PAGMA hybrid vesicles under different conditions: no stimulus (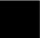), non-hybrid vesicles upon a 45 μM H_2_S stimulus (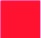), non-hybrid vesicles upon a 15 μM H_2_S stimulus (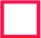), hybrid vesicles upon a 45 μM Cys stimulus (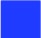) and hybrid vesicles upon a 15 μM Cys stimulus (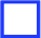). The polymer aggregate concentration is 0.02 g L^–1^, containing an AzMB group concentration of 45 μM.

In our experiments, the EP-loaded polymersomes were packed in a semipermeable bag (MWCO = 3.0 kDa), which was dialyzed against a phosphate buffer (pH = 7.2). The quantity of release was recorded through the intensity of EP diagnostic excitation^17^ (*λ*_ex_ = 317 nm) by fluorescence spectra. The release amount plotted against release time for various stimulation cases is depicted in Fig. 6. Without any trigger the nanocapsules showed a low-level free release process (less than 10% over 16 h). When applying a low concentration of H_2_S stimulus (15 μM), these vesicles expand but do not crack, thus leading to an increase of the number of membrane nanoporous defects in the vesicles. Such a result further enhances leaking of the internal EP payloads, so the release rate had a modest ascent (nearly 32% within 16 h). In contrast, when adding a high concentration of H_2_S stimulus (45 μM), the complete disruption of these polymer vesicles caused a rapid cargo release. The maximum release amount reached 94% in a shorter time (6 h).

In another case, we utilized Cys as an alternative stimulant to trigger the CSE-anchored hybrid polymersomes. In comparison to those corresponding entities without enzymes, their release speeds were decelerated (15 μM Cys caused a 25% release within 16 h and 45 μM Cys caused an 86% release within 10 h: blue dash line and blue solid line, respectively). The result that can be drawn is that our vesicles can serve as intelligent nanovehicles for realizing controllable drug delivery by modulating biosignal strength.

## Conclusions

In summary, we have designed and developed a new class of *o*-azidomethylbenzoate (AzMB)-containing block copolymers. They can spontaneously form a vesicular architecture in aqueous media on the basis of their amphiphilicity. The particular functionality in the polymer endows these vesicles with unique sensitivity to a gaseous signaling molecule, H_2_S. H_2_S can motivate a controlled disassembly of the polymersomes by site-specific cleavage of the AzMB groups. Moreover, we propose a concept, by means of installing various H_2_S-producing proteins onto the vesicle membrane, to reasonably extend the polymer responsive scope from simple H_2_S to complex biomolecules. With respect to drug delivery, due to this endogenous stimulus mode, the nanovectors are promising for intracellular targeted release and therapy. We envisage that this kind of polymer model will open up a new avenue to construct bioresponsive nanocapsules for more biological applications.

## Supplementary Material

Supplementary information
