# Supplementary material for: H2S gasotransmitter-responsive polymer vesicles
Source: Chem Sci. 2015 Dec 3;7(3):2100–5. doi: 10.1039/c5sc03576g (PMC5965249; doi:10.1039/c5sc03576g)
Supplement: Supplementary file 1 [file SC-007-C5SC03576G-s001.pdf]

Electronic Supplementary Material (ESI) for Chemical Science.
This journal is © The Royal Society of Chemistry 2015

Supplementary Information

H_2S gasotransmitter-responsive polymer vesicles

Qiang Yan and Wei Sang*

The State Key Laboratory of Molecular Engineering of Polymers, Department of Macromolecular Science, Fudan University, Handan Road 220, Shanghai 200433, P.R. China

Corresponding Author Email: yanq@fudan.edu.cn

1. Materials.

Methoxy poly(ethylene oxide) (PEO, $M_{n,GPC}$ = 3.0 kg/mol, M_w/M_n = 1.02) was dried by azeotropic distillation in the presence of toluene. 2-Bromo-isobutylryl bromide (BiBB, Sigma-Aldrich, 99%), triethylamine (TEA, Sigma-Aldrich, 98%), copper (I) bromide (CuBr, Sigma-Aldrich, 99.9%), pentamethyldiethylenetriamine (PMDETA, Acros, 99%) and indolin-1-one (Sigma-Aldrich, 99%) were used as received. 2-Azidomethyl benzoic acid was synthesized according to the literature.[1] The monomer of glycerol methacrylate (GMA, Alfa Aesar, 99%) was passed through a basic alumina column to remove the inhibitor for use. (±)-Epinephrine hydrochloride (EP, J&K Ltd., 97%) was used as received and dissolved in phosphate buffer (pH = 7.2) at a concentration of 0.10 mg mL^{-1}. Cystathionine γ-lyase (CSE), cysteine (Cys), methionine (Met), glutathione (GSH) and pyridoxal-5'-phosphate monohydrate (PLP) were purchased from Baoman Biotech. Ltd. Co. (Shanghai, China). All the solvent were used as received.

2. Characterization.

Nuclear Magnetic Resonance (NMR). ^{1}H NMR spectra for all the polymer samples were recorded by AVANCE III HD-400 (400 MHz) spectrometer with $CDCl_3$ or d_6-DMSO as the solvent.

Gel Permeation Chromatography (GPC). The molecular weight and the molecular weight distribution of all polymer samples were measured on a system of multiangle laser light scattering. The system is equipped with a Waters degasser, a Waters 515 HPLC pump, a 717 automatic sample injector, a Wyatt Optilab DSP differential refractometer, and a Wyatt miniDAWN detector. Three chromatographic columns (PLgel mix-H: 7.5×300 mm, PLgel guard-H: 7.5×50 mm, and Shodex GPC KD-806M: 8×300 mm) were used in series. HPLC-grade tetrahydrofuran (THF) was used as eluent at a flow rate of 1.0 mL/min at 30 °C and poly(ethylene oxide) as a standard reference.

Transmission Electron Microscopy (TEM). TEM images were measured on a Tecnai G2-20 TWIN instrument at a voltage of 120 kV. The specimen was prepared by drop-casting polymer solution onto carbon-coated grid and stained by 0.2%

phosphotungstic acid, finally freeze-drying before observation.

UV-Vis Spectroscopy (UV-Vis). The UV-Vis absorption spectra of the polymer samples at different H_2S stimulation conditions were recorded by using a Lambda-750 UV-Vis spectroscopy. When H_2S gas with various concentrations (0–40 μM) was exerted into the polymer solution, the samples were stirred for 20 min and incubated at room temperature for measurement.

Fluorescent Spectroscopy (FS). The critical aggregate concentration (CAC) of the PEO-*b*-PAGMA copolymer and the controlled drug release experiments using these polymer aggregates were conducted by an Edinburgh FLS920 fluorescent spectroscopy.

Dynamic Light Scattering (DLS). DLS experiment were performed on a Brookhaven goniometer (BI-200) equipped with a highly sensitive avalanche photodiode detector (Brookhaven, BI-APD), a digital correlator (Brookhaven, TurboCorr) that calculates the photon intensity autocorrelation function $g_2(t)$, a helium-neon laser (wavelength, λ = 632.8 nm), and a thermostat sample holder. The autocorrelation data was fitted using the CONTIN algorithm to determine the hydrodynamic radius of these polymer aggregates, and the change in scattering light intensity was measured at 90°.

Gas Injection. In all the gas experiments we employed commercial high-pure H_2S gas (Newrada Gas Ltd. Co., $H_2S \geq 99.9\%$, 8 L) as a gas source, and equipped with an electronic gas micro-flowmeter (Smit Instrument LWE-10, critical pressure: $P_c \leq 4.0$ MPa, flow rate: $0.01 \leq v \leq 150$ mL min^{-1}, $\pm\Delta v \leq 1.5\%$, operating temperature: -30 ^oC–80 ^oC) as a gaseous flow controller. When we added H_2S gas into the polymer systems, we fixed the gas flow rate at 0.1 mL min^{-1} and modulate the aeration time to control the total molar amount of H_2S gas. To further measure the concentration of H_2S in polymer solution, we also designed a set of gas collection instrument to calculate the amount of unreactive or leaked H_2S gas. The brief sketch of this equipment depicted as follows:

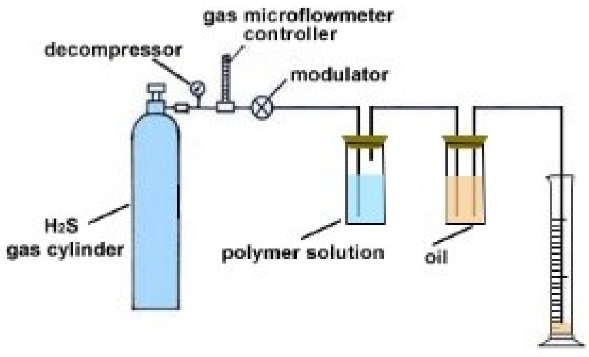

H_2S gas (0.1 MPa) was first passed through a decompressor for reducing pressure and flow rate, and then we tuned the flowmeter controller to obtain a required constant flow rate (0.1 mL/min). According to different conditions, we changed the aeration time and injected gas into our polymer solution for inducing chemical reactions. The total H_2S concentration (C_{total}) can be obtained from flow rate, aeration time and the

volume of our polymer solution. In fact, not all of gas can react with the polymer and a small amount of gas will leak out, we calculated this part of leaked gas via draining oil method (C_{leak}). Finally, we could obtain the actual reactive H_2S concentration with our polymer by the formula of $C_{reactive} = C_{total} - C_{leak}$. The oil collection was finished by high-precision miniature measuring cylinder (Sujing Lab Apparatus Co., V_{total}=1.0 mL, scale division is 0.01 mL, error volume is ±0.01 mL, the outside and inside diameter of the cylinder is 7.0 and 2.8 mm respectively, and the height is 145 mm). Using this high-precision measuring cylinder can obtain the volume of the excluded oil.

3. Polymer synthetic route.

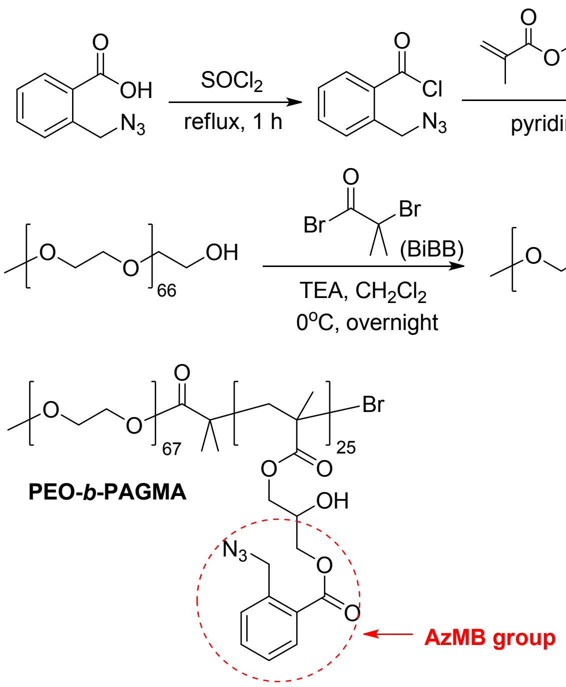

Scheme S1. The synthetic route of H_2S-responsive monomer *o*-azidomethylbenzoyl glycerol methacrylate (AGMA) and block copolymer poly(ethylene oxide)-*b*-poly(*o*-azidomethylbenzoyl glycerol methacrylate) (PEO-*b*-PAGMA) via ATRP protocol.

Synthesis of H_2S-sensitive monomer (AGMA).

A typical reaction was as follows: 2-Azidomethyl benzoic acid (3.542 g, 0.02 mol) was dissolved in 1.25 equiv. thionyl chloride ($SOCl_2$, 3.570 g, 0.03 mol) and reacted in neat under reflux for 1 h, followed by evaporation under reduced pressure. The acid chloride was used in situ since all attempts to purify it caused considerable decomposition. The resulted compound (3.51 g, yield ~90%) was reacted with glycerol methacrylate (GMA, 2.645 g, 0.016 mol) in pyridine (50 mL). The mixture was stirred at room temperature for 4 h, and then the crude products were distilled by reduced pressure to remove the excess of pyridine. Afterward the solids were dissolved in CH_2Cl_2 and poured into water with vigorous stirring for 1 h. The organic phase was collected and the water phase was extracted twice using CH_2Cl_2. The combined organic solution was further washed with 1.0 M $NaHCO_3$ solution, dried

over anhydrous $MgSO_4$ overnight and the solvent was then removed by reduced distillation. The product was dissolved in 2 mL of CH_2Cl_2 and purified by column chromatography with ethyl acetate/CH_2Cl_2 (5/1, v/v). Yield: 3.164 g, 62%. ^{1}H NMR (δ, ppm, $CDCl_3$): 7.72 (1H, d, in benzene group), 7.54 (1H, t, in benzene group), 7.34 (2H, m, in benzene group), 6.43 (1H, s, C***H***$_2$=CH(CH_3) COO-), 6.21 (1H, s, C***H***$_2$=CH(CH_3)COO-), 4.65 (1H, m, -COOCH$_2$C***H***(OH)CH$_2$O-), 4.42 (2H, t, -COOC***H*$_2$**CH-), 4.28 (2H, m, COOCH$_2$CH(OH)C***H*$_2$**O-), 3.42 (2H, s, -C***H*$_2$**N$_3$), 2.13 (3H, s, CH$_2$=CH(C***H*$_3$**)COO-). ^{13}C NMR (δ, ppm, $CDCl_3$): 168.9, 167.2 (two carbonyl), 136.5, 133.4, 129.4, 128.2, 125.4, 122.1 (in benzene group), 139.7 (CH$_2$=***C***H(CH$_3$)COO-), 124.0 (***C***H$_2$=CH(CH$_3$)COO-), 69.6 (-COOCH$_2$***C***H(OH)CH$_2$-O-), 67.1 (-COO***C***H$_2$CH(OH)CH$_2$O-), 66.3 (-COOCH$_2$CH(OH)***C***H$_2$O-), 59.5 (-***C***H$_2$N$_3$), 19.1 (CH$_2$=CH(***C***H$_3$)COO-).

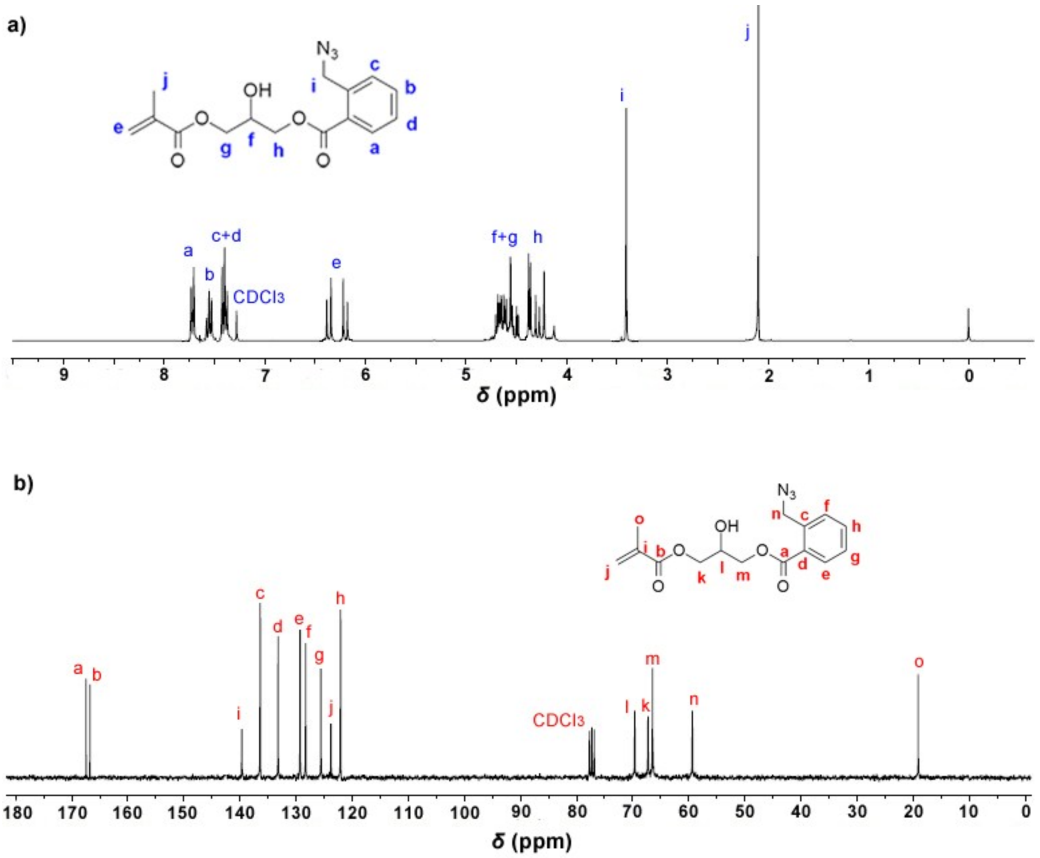

Fig. S1. (a) ^{1}H NMR and (b) ^{13}C NMR spectra of H_2S-sensitive AGMA monomer ($CDCl_3$ as solvent).

Synthesis of poly(ethylene oxide)-based macro-initiator (PEO-Br).

The PEO-Br macro-initiator was prepared by the reaction of PEO with excess of 2-bromo-isobutylryl bromide (BiBB) in the presence of triethylamine (TEA) according to the previous literature.[2] First, anhydrous PEO (3.000 g, 1.0 mmol) was dissolved in 60 mL of CH_2Cl_2, followed by adding TEA (0.202 g, 2.0 mmol). BiBB (0.570 g, 2.5 mmol, 2.5 equiv.) dissolved in anhydrous CH_2Cl_2 (20 mL) was finally injected dropwise over 2 h to the solution at 0 ^{o}C. Then the solution was stirred for 24 h and treated with activated charcoal. After filtration, the filtrate was collected and the

solvent was removed by rotary evaporation. The crude product was dissolved in CH_2Cl_2 and then poured into $NaHCO_3$ aqueous solution for 1 h. The extracted organic phase was further washed with 1.0 M HCl (10 mL), 1.0 M NaOH (10 mL) and deionized water (2×10 mL) successively, dried over anhydrous $MgSO_4$ overnight and the solvent was then removed by reduced distillation. The product was dissolved in 10 mL CH_2Cl_2 and precipitated into 100 mL of cold diethyl ether. This dissolution/precipitation cycles was repeated twice and obtained the solid. Yield: 2.810 g, 86%. $M_{n,\,NMR}$ = 3.16 kDa, $M_{n,GPC}$ = 3.12 kDa, M_w/M_n = 1.04. FT-IR (KBr, cm^{-1}): 2925 ($\nu_{C\text{-}H}$ in alkyl), 1726 ($\nu_{C=O}$ in ester group), 1152 ($\nu_{C\text{-}O\text{-}C}$ in PEO chain). ^1H NMR (δ, ppm, $CDCl_3$): 3.64 (s, -C$\boldsymbol{H}_2$C$\boldsymbol{H}_2$O-), 3.38 (s, -OC$\boldsymbol{H}_3$), 1.92 (s, -C(C$\boldsymbol{H}_3$)$_2$Br).

*Synthesis of poly(ethylene oxide)-b-poly(o-azidomethylbenzoyl glycerol methacrylate) via atom transfer radical polymerization (**PEO-b-PAGMA**).*

PEO-Br macro-initiator (0.325 g, 0.1 mmol), the monomer of **AGMA** (1.914 g, 6.0 mmol), CuBr (14 mg, 0.1 mmol), PMDETA (20 μL, 0.1 mmol), and 5.0 mL of anhydrous THF were added into a round bottom flask, followed by three freeze-vacuum-thaw cycles. The flask was reacted at 75 °C with magnetic stirring. After reaction for 8 h, the flask was immersed to liquid nitrogen in order to stop the radical polymerization. Then the solution was diluted to 30 mL of THF and passed through a neutral alumina column twice to remove the copper catalysts. The filtrate was concentrated to ~8 mL, and then precipitated into 300 mL of cold diethyl ether/methanol (1/1, v/v) mixture for three times. The product was collected and dried in vacuum oven at 25 °C for 24 h, yielding a white solid of 0.980 g (conversion: 35%). $M_{n,\,NMR}$ = 11.9 kDa, $M_{n,GPC}$ = 11.2 kDa, M_w/M_n = 1.09. FT-IR (KBr, cm^{-1}): 2922 ($\nu_{C\text{-}H}$ in alkyl), 2102 ($\nu_{N=N=N}$ azido group in AzMB), 1724 ($\nu_{C=O}$, ester group in main chain), 1614 (aromatic group in AzMB), 1152 ($\nu_{C\text{-}O\text{-}C}$ in PEO chain). ^1H NMR (δ, ppm, $CDCl_3$): 7.76 (d, benzene in AzMB), 7.57 (t, benzene in AzMB), 7.35 (m, benzene in AzMB), 4.63 (m, -COOCH$_2$C$\boldsymbol{H}$(OH)C$\boldsymbol{H}_2$O-), 4.43 (m, -COOCH$_2$C$\boldsymbol{H}$(OH)CH$_2$O-), 4.31 (t, -COOC$\boldsymbol{H}_2$CH-), 3.95 (s, methylene group in AzMB), 3.64 (s, -C$\boldsymbol{H}_2$C$\boldsymbol{H}_2$O- in PEO block), 3.10 (br, hydroxyl group), 1.26—1.58 (br, -[C$\boldsymbol{H}_2$C$\boldsymbol{H}$(CH$_3$)]$_n$- in PAGMA block), 1.13 (s, -[CH$_2$CH(C$\boldsymbol{H}_3$)]$_n$- in PAGMA block.

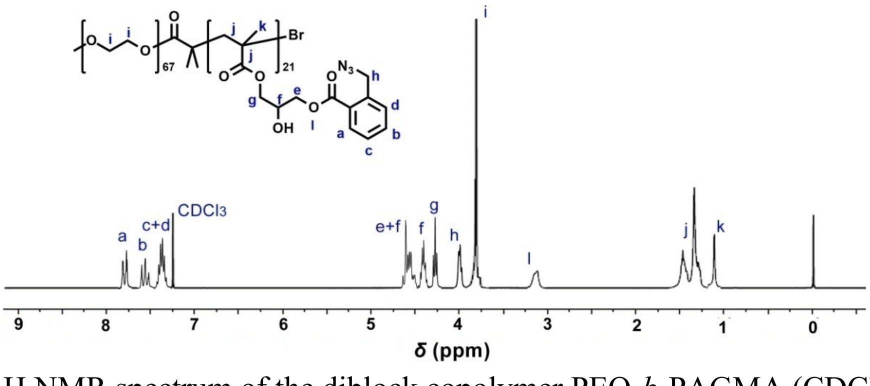

Fig. S2. ^1H NMR spectrum of the diblock copolymer PEO-*b*-PAGMA ($CDCl_3$ as solvent)

4. H_2S-induced cascade reaction of PEO-*b*-PAGMA copolymer

When we added H_2S (20 μM) into the PEO-*b*-PAGMA copolymer solution (9×10^{-3} g L^{-1}, containing ~20 μM AzMB group), the polymer began to remove the AzMB groups to yield PEO-*b*-PGMA, and the solution generated a new kind of cyclic benzolactam byproduct. First, we used NMR spectroscopy to survey whether PEO-*b*-PAGMA can be decomposed to PEO-*b*-PGMA by H_2S treatment. As shown in ***Fig. S3a***, we designed and synthesized a water-soluble diblock copolymer PEO_{67}-*b*-$PGMA_{35}$, and it showed typical glycerol group proton peaks [δ = 3.82 (t), 4.26 (m) and 4.63 (m)]. As compared to the decomposed product by H_2S gas (***Fig. S3b***), its NMR proton shifts are almost consistent with that of PEO_{67}-*b*-$PGMA_{35}$. Thus, we can deduce that H_2S can cleave the AzMB groups and convert PEO-*b*-PAGMA to water-soluble PEO-*b*-PGMA.

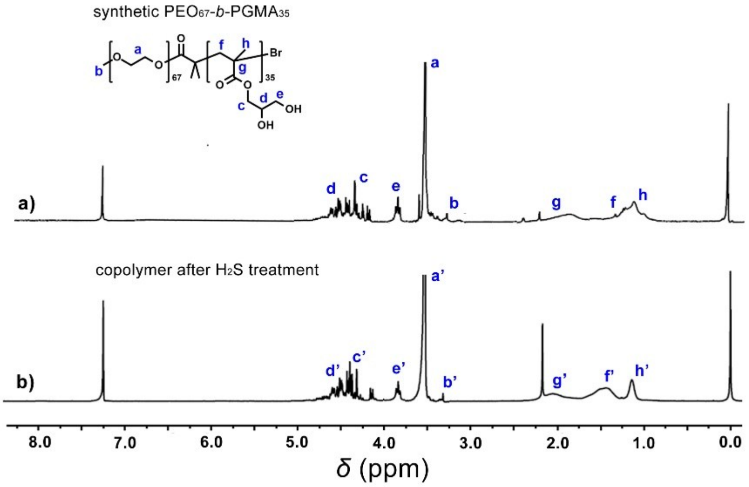

Fig. S3. ^{1}H NMR spectral comparison of (a) synthetic PEO_{67}-*b*-$PGMA_{35}$ and (b) H_2S-decomposed polymer product of PEO_{67}-*b*-$PAGMA_{21}$

Next, according to the H_2S-induced cascade reaction as Eq.(1),[1] we deduced that the byproduct is possible to be indonlin-1-one. To survey this mechanism, we used standard indonlin-1-one (purchased from Sigma-Aldrich) as a reference and carried out control experiments by means of NMR and UV-Vis spectroscopy.

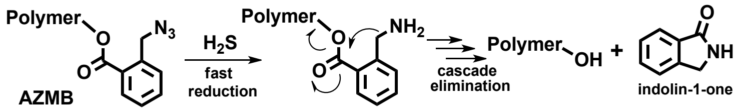

..............(1)

As shown in ***Fig. S4a***, the standard indonlin-1-one showed a series of acrylic group proton resonances [δ = 7.89 (t, 1H), 7.76 (m, 1H), 7.41 (t, 1H), 7.20 (m, 1H)], methylene peak [δ = 4.18 (s, 2H)] and acrylamide peak [(δ = 3.82 (br, 1H)]. In the other hand, after we applied H_2S gas stimulus to the PEO-*b*-PAGMA, the decomposed copolymer was collected by precipitation method. Except for the copolymer, there was still a kind of unknown byproduct existing in the filtrate. As we purified this byproduct, its ^{1}H NMR spectrum (***Fig. S4b***) was found to be consistent with that of the indonlin-1-one, which indicates that low concentration of H_2S stimulus can induce our copolymer to undergo the cascade reactions for removing the AzMB groups and further forming the PEO-*b*-PGMA copolymer.

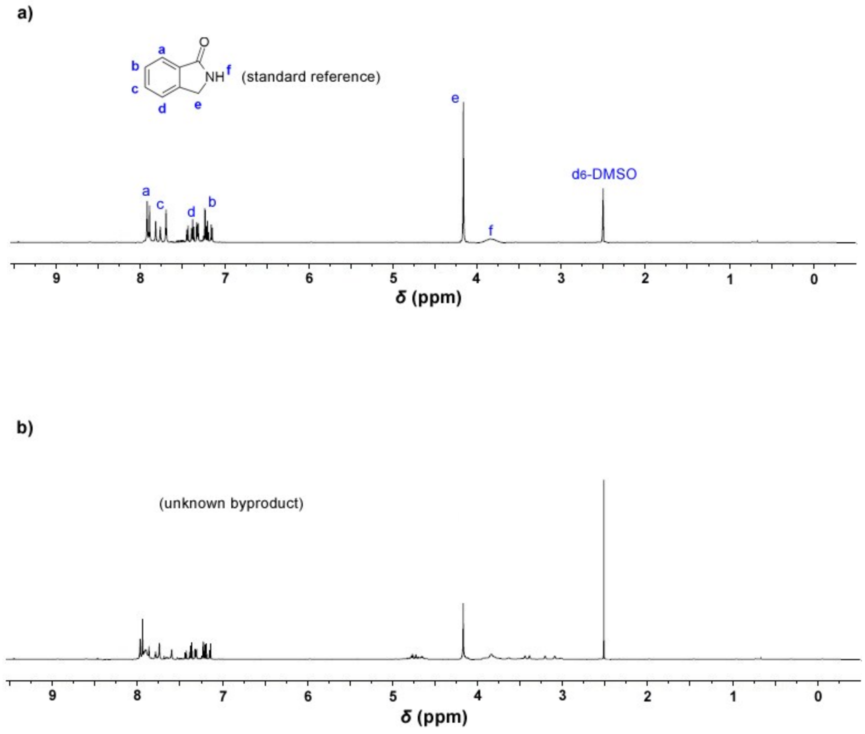

Fig. S4. (a) ^{1}H NMR spectrum of the standard indonlin-1-one reference, (b) ^{1}H NMR spectrum of the unknown byproduct existing in the solution after H_2S treatment (d_6-DMSO as solvent)

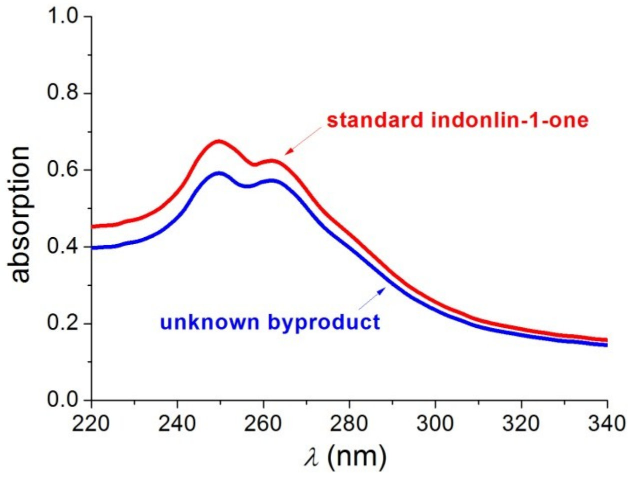

Fig. S5. The UV-Vis absorption comparison between standard indonlin-1-one (red line) and the unknown byproduct after H_2S-induced chemical reaction (blue line). The standard indonlin-1-one sample is dissolved in DMSO solvent at a concentration of 2.0×10^{-6} M and the byproduct was first purified and then dissolved in DMSO at the similar concentration.

Furthermore, we employed UV-Vis spectroscopy to monitor the difference between the standard indonlin-1-one and the byproduct from H_2S-sensitive decomposition

reaction. As shown in ***Fig. S5***, it is noted that standard indonlin-1-one displayed a double-peak absorption at λ = 248 and 259 nm (red curve), which is consistent with that of the decomposed unknown byproduct (blue curve). This result further confirmed the above conclusion that H_2S can induce a cascade reaction and cut off the PEO-*b*-PAGMA to yield indonlin-1-one, like the reaction process as Eq. (1).

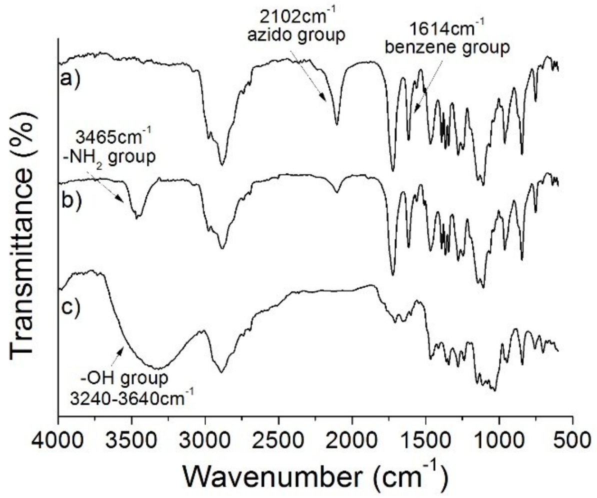

Fig. S6. FT-IR spectra of the PEO-*b*-PAGMA in different stimulus conditions: (a) no stimulus, (b) 6 min of H_2S stimulus and (c) 30 min of H_2S stimulus.

At last, we also employed FT-IR spectroscopy to survey the spectral changes of the polymer system before and after H_2S gas treatment. In the IR experiments, we fixed the H_2S gas flow rate at 0.1 mL min^{-1} and modulated the aeration time from 0 to 30 min. We prepare three copies of PEO-*b*-PAGMA solution (THF/H_2O, 5/1, 0.2 g L^{-1}, 10 mL) for H_2S stimulation. The first sample was in the absence of H_2S and removed the solvent by lyophilization for obtaining solid sample to perform IR characterization. The second sample was passed through H_2S for 6 min, and then stopped the gas flow and removed the solvent to gain solids for IR characterization. The third one was injected H_2S gas for 30 min, and then removed the solvent to characterize. Before H_2S stimulus, it is clear that the PEO-*b*-PAGMA showed typical symmetric stretching vibration of benzene ring and azido group at 1614 cm^{-1} and 2102 cm^{-1}, respectively (***Fig. S6a***). However, after H_2S has passed through the polymer system for 6 min, the vibration band ascribed to azido group was strongly depressed but the band of aromatic group kept constant, concomitantly, a new shoulder peak at 3465 cm^{-1} ascribed to the typical benzylamine group appeared (***Fig. S6b***). It is demonstrated that at the early stage of this reaction, the azido group can be fast reduced by H_2S to convert into benzylamine group. Further prolonging the H_2S stimulation time to 30 min, the three vibration bands of benzylamine group, azido group and aromatic group all vanished, whereas a broad absorption band belonged to hydroxyl group (3240–3640 cm^{-1}) was strengthened (***Fig. S6c***), which results from the removal of AzMB group from the polymer main chain. These findings confirmed that H_2S can fast transform benzylazide into a high-reactive nucleophilic benzylamine intermediate, and the latter is capable of attacking intramolecularly on the adjacent benzoyl to

induce cascade self-elimination reaction, leading to a site-specific chemical scission.

5. PEO-*b*-PAGMA copolymers self-assemble in aqueous solution

5.1 Critical aggregate concentration (CAC) of the PEO-b-PAGMA in aqueous solution

A 5 mL solution of 2.0 g L^{-1} PEO-*b*-PAGMA in THF was added into 5 mL of deionized water under sonication. Then the solution followed by dialysis against deionized water to obtain an aggregate solution at a concentration of 1.0 g L^{-1} for further experiments. The critical aggregate concentration (CAC) of PEO-*b*-PAGMA was tested by pyrene fluorescent probe method. A 10 μL of 5×10^{-5} g L^{-1} pyrene solution in acetone was mixed with the polymer to obtain a series of PEO-*b*-PAGMA/pyrene aqueous solutions with different concentrations (1×10^{-3}, 2×10^{-3}, 5×10^{-3}, 0.01, 0.02, 0.05, 0.1, 0.2, 0.5 and 0.75 g L^{-1}) and the solutions were sonication for 10 min before fluorescent measurements. The results exhibited that the CAC is about 0.02 g L^{-1} (The CAC was chosen as the concentration when pyrene probe showed an apparent decrease in the I_1/I_3 ratio with an increasing concentration of the copolymer, indicating that the aggregation occurred, ***Fig. S7***).[3]

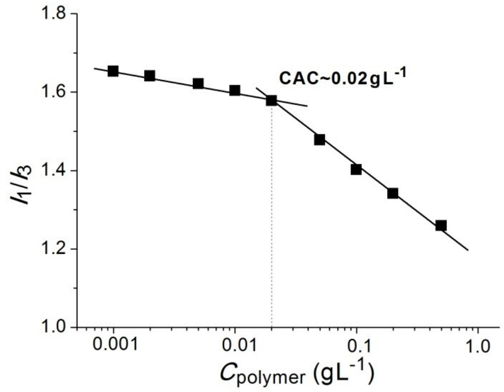

Fig. S7. The CAC of the copolymer PEO-*b*-PAGMA by using the fluorescent method and the CAC is determined to be ~0.02 g/L in aqueous media.

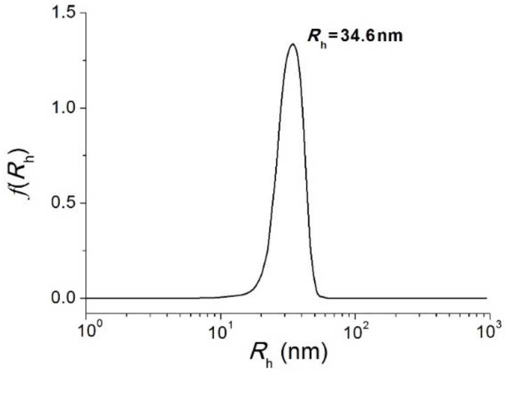

Fig. S8. The hydrodynamic radius (R_h) of the PEO-*b*-PAGMA aggregates in aqueous solution is 34.6 nm without any external stimuli.

5.2 The hydrodynamic radius of the PEO-b-PAGMA vesicles in aqueous solution

When the PEO-*b*-PAGMA copolymer dissolved in aqueous media, in the absence of trigger, they can self-assemble into aggregated nanostructures. Dynamic light scattering (DLS) analysis showed that the hydrodynamic radius (R_h) of these polymer aggregates is about 34.6 nm (***Fig. S8***).

6. The copolymer counterpart

From all the experimental results, we found that the PEO-*b*-PAGMA vesicles could be completely disassembled by H_2S gas stimulation. Besides the mechanism of H_2S-induced polymer cleavage, mechanical explosion effect driven by gas bubbles might be another possible explanation for this vesicular disruption phenomenon. To eliminate this possibility, we synthesized a copolymer counterpart, PEO-*b*-poly(benzoyl glycerol methacrylate) (PEO-*b*-PBGMA). PEO-*b*-PBGMA has a similar chain structure to PEO-*b*-PAGMA but is lack of the azido group (***Fig. S9a***). In aqueous solution, they can also self-assemble into analogous vesicular morphology with the average diameter of 74 nm. However, when we exerted H_2S gas (100 μM) to the PEO-*b*-PBGMA aggregate solution, these assemblies had no obvious changes either in size or morphology (***Fig. S9b—c***), which indicates that H_2S is unable to dissociate PEO-*b*-PBGMA vesicles. Thereby, this result eliminates the possibility of mechanical explosion effect and further supports that the disassembly mechanism of our polymer vesicles arises from H_2S-sensitive polymer structural alteration.

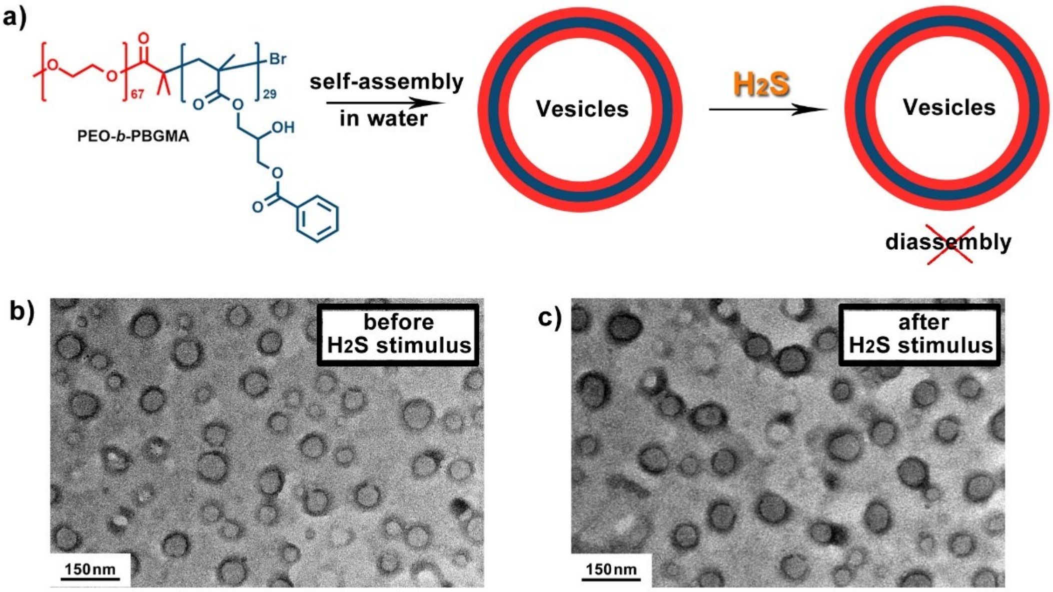

Fig. S9. (a) Schematic representation of the self-assembly process of the copolymer counterpart PEO-*b*-PBGMA and the H_2S-insensitivity of their aggregates; TEM images of the PEO-*b*-PBGMA vesicles before (b) and after (c) H_2S gas treatment (The polymer concentration is kept at 0.02 g L^{-1} as same as the PEO-*b*-PAGMA copolymer solution)

7. H_2S-Responsive Specificity of PEO-*b*-PAGMA Copolymer

We have demonstrated that our PEO-*b*-PAGMA polymersomes possess H_2S-triggered disassembly ability. Because we expected that these responsive nanocapsules could be applied in biological cells, thus these polymersomes should possess high-specificity to intracellular H_2S neurotransmitter. However, besides H_2S signaling molecule there are other sulphur-containing bioactivators in cell such as cysteine (Cys), methionine (Met) and glutathione (GSH).[4] To detect whether they have similar responsiveness, we used UV-Vis spectroscopy to monitor their reactivity to our PEO-*b*-PAGMA copolymer. As shown in ***Fig. S10a***, we found that H_2S causes the decomposition of PEO-*b*-PAGMA and the reactive byproduct, indolin-1-one, led to a remarkable change in UV-Vis spectra. In a similar way, when we added other biological stimulants into our polymer system (stimulant concentration is 45 μM and the treatment time keep in 60 min), respectively, their solution UV-Vis spectra had negligible changes (***Fig. S10b***, Cys; ***Fig. S10c***, Met; ***Fig. S10d***, GSH). These confirmed that these sulphur-containing bioactivators have no ability to induce the polymer vesicle disassembly. These results demonstrate that our polymersomes are of high-specific H_2S-responsiveness.

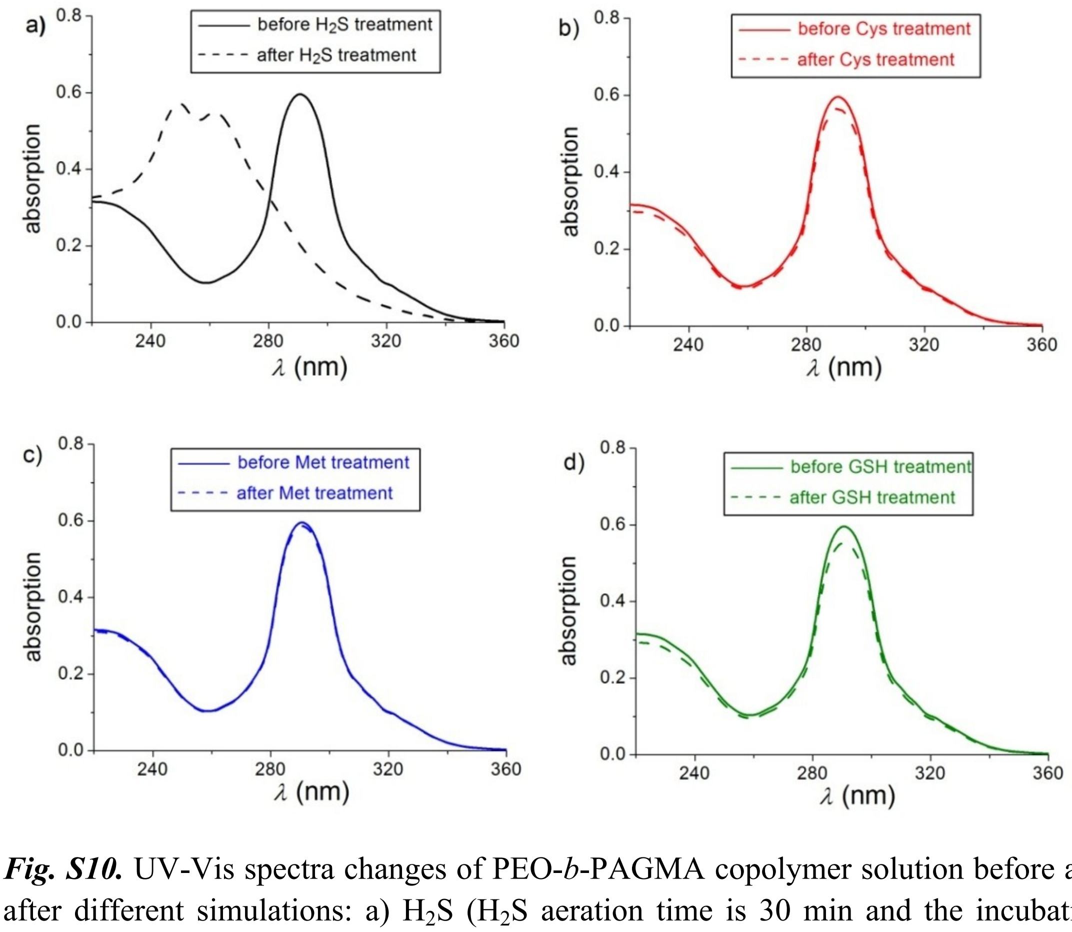

Fig. S10. UV-Vis spectra changes of PEO-*b*-PAGMA copolymer solution before and after different simulations: a) H_2S (H_2S aeration time is 30 min and the incubation time kept in 30 min), b) Cys, c) Met, and d) GSH (all of the bioactivator concentration is kept at 45 μM and treatment time is kept in 60 min).

8. CSE Enzyme Co-assembled with the Polymer Vesicles

To further extend the responsive scope of our polymer vesicles, we attempted to anchor CSE enzyme onto the vesicular membrane. First, we adopted a thin film-rehydration method to co-assemble the CSE enzyme with our polymer vesicles: 0.2 mg of the PEO-*b*-PAGMA copolymer was dissolved in 5 mL of THF placed into a 25 mL round-bottom flask, and then the organic solvent was removed to form a dry film by rotary evaporation. After purging with N_2 for 15 min, 20 nM of CSE enzyme solution (PBS buffer, 10 mL) was added into the flask using a syringe. The mixing solution was stirred overnight to ensure the self-assembly process. The CSE-anchored vesicles were separated out as the precipitant by ultracentrifugation at 11000 rpm for 20 min. Then the precipitant was re-dispersed in PBS buffer (10 mL) to form hybrid assemblies.

After the vesicle formed, there should be a part of CSE residue dispersed in the solution. To remove these residues, the aggregate solution was centrifuged for 5 min at 11000 rpm at certain intervals (2 h), and 2 mL of the supernatant was withdrawn and replaced by fresh medium. The CSE residue was existed in the supernatant and was assayed via absorbance at typical 469 nm in UV-Vis spectrum. Repeating this centrifugation process ensures that the supernatant had no obvious absorption, which indicates that the CSE residue was removed from the vesicle solution. According to the established calibration curve, there were ~35% CSE enzyme associated with the PEO-*b*-PAGMA vesicles.

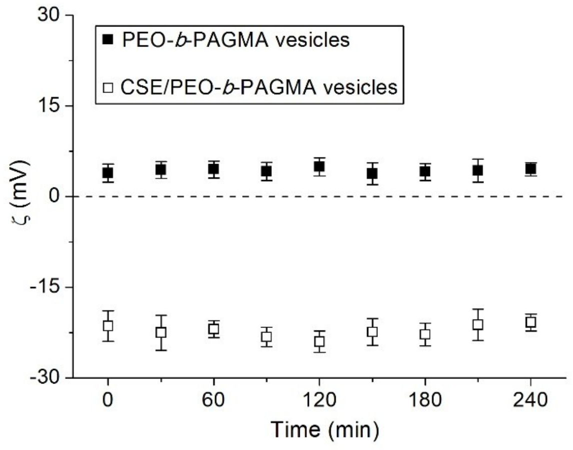

Fig. S11. The surface zeta-potential change of the polymer aggregate during 6 h (black square: pure PEO-*b*-PAGMA vesicles; hollow black square: CSE enzyme co-assemble with PEO-*b*-PAGMA copolymer to form hybrid vesicles).

Finally, to further confirm that CSE enzyme was anchored onto the vesicle membrane surface, we employed zeta-potentiometer to monitor the surface charge of the polymer aggregate before and after the addition of CSE enzyme. As shown in **Fig. S11**, the pure PEO-*b*-PAGMA vesicles exhibited a low positive potential (+4 mV, black square). However, since the CSE protein is a kind of enzyme with negative surface charges, the surface potential of the CSE-polymer hybrid vesicles changed from +4 mV to -22 mV (hollow black square), which indicates that the CSE enzyme can be

anchored onto the polymer vesicular membrane.

References

[1] T. Wada, A. Ohkubo, A. Mochizuki and M. Sekine, *Tetrahedron Lett.,* **2001**, 42, 1069-1072.
[2] J. Z. Du, Y. M. Chen, *Macromolecules,* **2004**, 37, 6322-6328.
[3] A. Mohr, P. Talbiersky, H.-G. Korth, R. Sustmann, R. Boese, D. Bläser and H. Rehage, *J. Phys. Chem. B.*, **2007**, 111, 12985-12992.
[4] J. T. Brosnan, M. E. Borsnan, *J. Nutr.,* **2006**, 136, 1636S-1640S
